# Lactate thresholds and role of nitric oxide in male rats performing a test with forced swimming to exhaustion

**DOI:** 10.14814/phy2.15801

**Published:** 2023-09-04

**Authors:** Natalya Potolitsyna, Olga Parshukova, Nadezhda Vakhnina, Nadezhda Alisultanova, Lubov Kalikova, Anastasia Tretyakova, Alexey Chernykh, Vera Shadrina, Arina Duryagina, Evgeny Bojko

**Affiliations:** ^1^ Institute of Physiology of Kоmi Science Centre of the Ural Branch of the Russian Academy of Sciences, FRC Komi SC UB RAS Syktyvkar Russia

**Keywords:** anaerobic threshold, exhaustion, lactate biochemical indices, rats, swimming test

## Abstract

The present study assessed a complex of biochemical parameters at the anaerobic threshold (AT) in untrained male Wistar rats with different times to exhaustion (T_ex_) from swimming. The first group of rats was randomly divided into six subgroups and subjected to a swimming test to exhaustion without a load or with a load of 2%–10% of body weight (BW). In the first group, we established that for untrained rats, the load of 4% BW in the swimming to exhaustion test was optimal for endurance assessment in comparison with other loads. The second group of rats went through a preliminary test with swimming to exhaustion at 4% BW and was then divided into two subgroups: long swimming time (LST, T_ex_ > 240 min) and short swimming time (SST, T_ex_ < 90 min). All rats of the second group performed, for 6 days, an experimental training protocol: swimming for 20 min each day with weight increasing each day. We established that the AT was 3% BW in SST rats and 5% BW in LST rats. The AT shifted to the right on the lactate curve in LST rats. Also, at the AT in the LST rats, we found significantly lower levels of blood lactate, cortisol, and NO.

## 1 INTRODUCTION

An important practical challenge in modern sports is to obtain objective information on an athlete's physical performance and fitness during the training process. One of the most informative and popular methods for assessing the functional status of athletes is to study the anaerobic threshold (AT) (Ghosh, 2004; Solli et al., 2017). The AT represents the transition to an anaerobic mechanism of energy exchange when performing physical activity at submaximal and maximum power. The production and excretion of blood lactate are in equilibrium at the AT (MLSS, maximal lactate steady state), and a significant increase in the lactate level is observed (i.e., the lactate threshold) then the athlete transitions through the AT zone (Abreu et al., 2016; Gobatto et al., 2001). Despite numerous studies, the metabolic basis of AT has not been fully established. Studies of AT in humans are often complicated by the infeasibility of complete control over the experiments, and in‐depth deep, long‐term and high‐intensity studies of the AT phenomenon are not always possible in humans (Cholewa et al., 2014). Therefore, to understand many fundamental aspects of exercise physiology, research using an adequate animal model is needed. Rodents are among the most popular and easily available laboratory animals for use in such models. Compared to human studies, working with animals allows for greater control and regulation of environmental conditions and food intake. These studies make it possible to collect different types of tissues and perform a number of experimental manipulations that cannot be performed in humans (Cholewa et al., 2014). The results of studies on rats are traditionally projected onto humans (Voltarelli et al., 2002). There is evidence that rats adequately reflect the human response to physical activity based on the main biochemical parameters of blood (Goutianos et al., 2015). However, rodents and humans may not have similar reactions to physical exercise (Greek et al., 2012; Rice, 2012).

The AT in animals is determined by various changes in blood biochemistry (Faude et al., 2009):

1. when animals achieve the reference lactate level in the blood (4 mmol/L) during exercise (Heck et al., 1985);
2. when the first increase in blood lactate levels above the baseline level is detected (onset of blood lactate accumulation) (Farrell et al., 2018; Faude et al., 2009);
3. when a notable bend (sharp change in curvature) in the lactate curve caused by massive lactate accumulation during physical load is observed (Contarteze et al., 2008; Gobatto et al., 2001);
4. when undergoing a short period of submaximal load to induce hyperlactemia before starting the test with an increasing load (lactate minimum test, LMT) (Voltarelli et al., 2002).

In our opinion, fixed or precalculated lactate levels might not take into account considerable interindividual differences and differences between various lactate analyzers (Faude et al., 2009). Therefore, the most objective AT assessment method is by observing a sharp change (bend) in the lactate curve during exercise with an increasing load. Then, the AT is determined as the physical load equivalent to MLSS; in other words, the highest exercise load at which the lactate levels in blood do not change significantly (Contarteze et al., 2008; Faude et al., 2009; Heck et al., 1985). In the case of individual lactate curves, visual curve assessment is used. For groups, we believe a statistically significant increase in blood lactate levels in comparison with the previous physical load is more appropriate.

It is also important to monitor other biochemical indicators that characterize the level of physiological adaptations of the body during exercise. Indicators in sports physiology, such as cortisol, catecholamines, glucose, urea, and other metabolites, are most often used (De Araujo et al., 2016; Halson & Jeukendrup, 2004). Various studies have also proposed other markers of AT, including blood catecholamines (Davies et al., 1974) and saliva amylase (Chicharro et al., 1999). We showed that elite athletes (cross‐country skiers) had a nitric oxide‐dependent (NO‐dependent) mechanism for regulating lactate levels during aerobic exercise, especially when working at the AT. In our previous work, we revealed a positive relationship between NO metabolites and blood lactate at the AT, which was reversed at maximum load. This observation suggests the existence of an adaptive mechanism for regulating the level of lactate on the AT in highly qualified cross‐country skiers (Parshukova et al., 2022).

The time of onset of the anaerobic (lactate) threshold largely depends on the duration of the load (Papadopoulos et al., 2006; Roecker et al., 1998; Weyand et al., 1994), physical fitness level (Støren et al., 2014; Tanji & Nabekura, 2019), intensity of movement (Wakayoshi et al., 1993), testing methods (Contarteze et al., 2008; De Araujo et al., 2016), and swimming patterns in swimmers (Dos Reis et al., 2018). Perhaps due to the influence of a large number of factors, research results are often contradictory. Therefore, doubts are expressed that the AT (as an indirect method for determining aerobic endurance) reflects the optimal intensity of training, especially for elite athletes (Bosquet et al., 2002). Determination of the duration of exercise to exhaustion is a direct method for determining aerobic endurance and provides more complete and reliable information (Beck et al., 2014). There are few studies of the AT with measurements of the maximum duration of physical activity to exhaustion in untrained rats.

Therefore, the hypothesis of the current study was that the characteristics of the AT will be different in rats displaying different times to exhaustion (T_ex_) while performing the same swimming tests in the same conditions. The purpose of our study was to assess the biochemical parameters at the AT in untrained rats with different endurance levels performing a swimming test to exhaustion.

## 2 MATERIALS AND METHODS

### 2.1 Experimental animals

Our study used male Wistar rats (*n* = 60), aged 8 weeks at the beginning of the experiment, weighing 250–300 g. Rats were housed in a room with a temperature of 21 ± 1°C and a controlled photoperiod (12 h of light/12 h of darkness) on a standard vivarium diet, with access to water ad libitum. The protocol of the study was reviewed for compliance with the “Rules of the European Convention for the Protection of Vertebrates Used for Experimental and Other Scientific Purposes” and approved by the local Ethics Committee of the Institute of Physiology of the Komi Research Center of the Ural Branch of the Russian Academy of Sciences.

### 2.2 Adaptation to water

Prior to experiments, all animals were adapted to water, with a subsequent recovery for 14 days (Brito et al., 2015). Adaptation consisted of keeping animals in shallow water at a temperature of 31 ± 1°C for 30 min for 14 days. The purpose of the adaptation period was to familiarize the animals with testing conditions while avoiding physical training adaptations.

### 2.3 Experimental procedures

The swimming sessions were performed in cylindrical water tanks (height 60 cm × diameter 45 cm) with a water temperature of 30 ± 1°C and indoor air temperature of 22 ± 1°C. The rats swam in individual tanks with desaturated water. After weighing the animals, a metal weight of the necessary mass was affixed to the base of the tail using elastic nontraumatic tape. A stopwatch was started when the animals were placed in the water. Exhaustion was defined as the animals being incapable of staying on the water surface, the loss of symmetrical movements during swimming, or the animals remaining underwater for more than 10 s (Chimin et al., 2013). No deaths occurred during or after exercise in any of the animal subgroups.

### 2.4 Method for assessing physical activity to exhaustion

Rats (*n* = 50) were randomly divided into six groups. Each group performed a swimming test to exhaustion with one of the following swim loads (SL): without load (SL0, *n* = 9) or with a load of 2% (SL2, *n* = 8), 4% (SL4, *n* = 8), 6% (SL6, *n* = 10), 8% (SL8, *n* = 8), and 10% (SL10, *n* = 7) of body weight (BW). After the animals achieved exhaustion, they were immediately removed from the water tank, dried, anesthetized, and sacrificed via decapitation.

### 2.5 Method of measuring the AT

**FIGURE 1:**
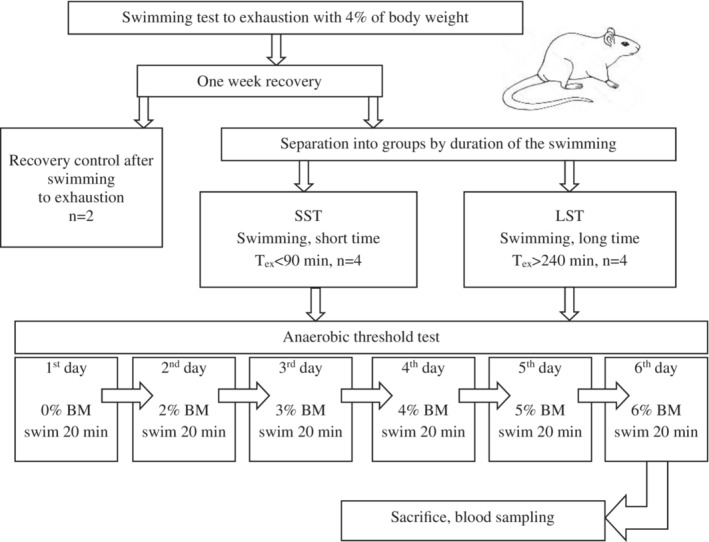
The protocol for anaerobic threshold evaluation in rats.

To determine the AT, we used the method of Gobatto et al. (2001) with modifications (Figure 1). A total of 10 rats were used in the study. All rats had previously gone through the test to exhaustion with a load of 4% BW. Based on this test, all rats were divided into two groups: rats with a long swimming time (time to exhaustion, T_ex_ > 240 min, LST, *n* = 5) and rats with a short swimming time (T_ex_ < 90 min, SST, *n* = 5). After this test, all rats were allowed to recover for 1 week. One rat from each group was decapitated to assess the recovery of the blood biochemistry parameters after exertion to exhaustion (recovery control after swimming to exhaustion). The remaining rats were subjected to physical activity to assess the AT. Each animal participated in six experimental tests over 6 days with a 24‐h interval between tests. Each test consisted of continuous swimming for 20 min with a load of 0%, 2%, 3%, 4%, 5%, or 6% BW in a tank filled with desaturated water at a temperature of 31 ± 1°C. Immediately after performing the exercise, the rats were removed from the water, and their tails were heated with warm water and dried with a towel. Blood samples were taken from the tail vein using a syringe and placed in heparinized Eppendorf tubes (1.5 mL capacity). After performing the test with last load (6% BW), the rats were sacrificed.

We determined the AT by observing a sharp increase in blood lactate using the lactate curve obtained by performing physical load tests (Faria et al., 2021; Faude et al., 2009). The increase between the two consecutive loads was required to be statistically significant. The AT in this case was the lower of the two consecutive physical load intensities, the one after which the increase was observed.

### 2.6 Blood samples and analyses

The lactate levels in the blood samples collected from the caudal veins were determined using a lactate analyzer (Accutrendplus, Roche Diagnostics GmbH). Blood (mixed, arteriovenous) after decapitation was collected in tubes containing heparin and centrifuged at 2400 rpm for 10 min at 4°C. The samples were frozen and stored at −40°C. Plasma levels of lactate (Sentinel Diagnostics), urea, glucose, and cortisol (all from Human GmbH) were measured using immunoenzyme assays (ChemWell 2900 biochemical analyzer). Levels of NO in the plasma were measured using the Griess reaction by evaluating stable metabolites of NO, including nitrites (NO_2_) and nitrates (NO_3_), which were merged together as an index (NO_x_). These methods were previously described (Parshukova et al., 2020, 2022).

### 2.7 Statistical analysis

All values are expressed as the means ± SD. Statistical analyses were performed using Statistica 8.0 (Statsoft). The statistical significance of differences between the SST and LST groups was estimated using the Mann–Whitney (*U*) test. For comparisons of multiple independent groups, we used the Kruskal–Wallis test. For comparisons between workload groups within a corresponding group, we used Friedman ANOVA and Kendall's coefficient of concordance. When necessary, the Newman–Keuls post hoc comparison test was used. The statistical significance level was set at *p* < 0.05.

## 3 RESULTS

The times of swimming to exhaustion and the parameters of blood biochemistry in rats performing the T_ex_ test with various loads are presented in Table 1.

**TABLE 1:** Swimming time and biochemical parameters of blood in untrained male rats (all group) after performing swimming tests to exhaustion with different loads.

Work load groups	Swimming time, min	Glucose, mmol/L	Lactate, mmol/L	Cortisol, ng/mL	Urea, mmol/L	NO_х_, μmol/L	NO_2_, μmol/L	NO_3_, μmol/L
SL0	419.7 ± 229.1	4.9 ± 1.5	4.1 ± 2.5	21.0 ± 3.3	8.0 ± 3.5	37.0 ± 21.8	5.2 ± 2.3	31.8 ± 21.7
	*SL6, SL8, SL10	*SL10	*SL8		*SL10			
SL2	148.6 ± 130.2	5.2 ± 2.3	6.8 ± 4.4	19.8 ± 8.3	5.3 ± 1.8	27.7 ± 13.5	9.1 ± 2.1	18.5 ± 13.9
		*SL4	*SL4			*SL4	*SL4	*SL4
SL4	89.1 ± 86.9	4.7 ± 2.4	5.7 ± 4.8	23.7 ± 5.5	5.1 ± 2.0	40.9 ± 8.2	3.9 ± 0.8	37.0 ± 7.8
		*SL2, SL10	*SL2, SL8		*SL10	*SL2, SL8	*SL2	*SL2, SL6, SL8, SL10
SL6	59.9 ± 83.1	6.0 ± 2.8	11.0 ± 6.7	23.0 ± 5.0	6.3 ± 1.4	22.1 ± 13.0	6.4 ± 3.0	15.7 ± 15.1
	*SL0							*SL4
SL8	3.0 ± 1.1	8.6 ± 2.9	14.2 ± 1.4	16.1 ± 2.4	5.0 ± 2.0	12.3 ± 2.5	5.6 ± 1.4	6.8 ± 2.7
	*SL0		*SL0, SL4			*SL4		*SL4
SL10	2.2 ± 0.4	9.8 ± 1.8	13.3 ± 1.3	16.5 ± 3.8	2.8 ± 1.1	14.1 ± 2.2	6.6 ± 1.9	7.5 ± 1.7
	*SL0	*SL0, SL4			*SL0, SL4			*SL4

*Note*: The values were expressed as means ± SD.

* The differences between workload groups are significant at <0.05 for the Kruskal–Wallis ANOVA test.

The total swimming time of rats performing the test to exhaustion expectedly decreased with increasing tail weights and showed significant variation between individual rats. The greatest variation between minimal and maximal times of swimming was observed in the group swimming with weights of 0%–6% BW. All rats within each group were clearly divided by the duration of swimming into LST and SST groups (Table 2). The duration of swimming did not exceed 4 min in the SL8 and SL10 groups, and these values did not differ significantly between the SL8 and SL10 groups.

**TABLE 2:** Swimming time and biochemical parameters of blood in short swimming time (SST) and long swimming time (LST) male rats after performing swimming tests to exhaustion with different loads.

Work load groups	Swimming time, min		Glucose, mmol/L		Lactate, mmol/L		Cortisol, ng/mL		Urea, mmol/L		NO_х_, μmol/L		NO_2_, μmol/L		NO_3_, μmol/L	
	SST	LST	SST	LST	SST	LST	SST	LST	SST	LST	SST	LST	SST	LST	SST	LST
SL0	168.0 ± 24.0	545.5 ± 163.5	4.9 ± 2.3	5.0 ± 1.3	7.2 ± 1.6	2.5 ± 0.7	24.5 ± 2.2	19.2 ± 2.0	7.2 ± 5.5	8.5 ± 2.6	23.0 ± 18.7	42.0 ± 8.2	5.8 ± 2.1	5.2 ± 2.4	17.3 ± 19.9	36.8 ± 19.5
	*SL6	#			*SL6	*SL6#		#								
SL2	48.0 ± 14.2	282.7 ± 58.0	6.4 ± 2.4	3.5 ± 0.2	10.3 ± 0.5	2.1 ± 0.6	25.6 ± 5.5	15.4 ± 3.7	4.3 ± 0.7	6.6 ± 2.1	20.0 ± 3.5	37.9 ± 11.1	10.0 ± 1.1	8.0 ± 2.9	10.1 ± 2.4	29.9 ± 15.4
	*SL6	#		#		#				#						#
SL4	29.2 ± 7.4	189.0 ± 48.8	5.8 ± 2.5	2.9 ± 0.5	8.3 ± 4.1	1.3 ± 0.2	26.0 ± 5.9	19.9 ± 1.0	4.7 ± 2.1	5.7 ± 2.0	37.0 ± 8.1	47.3 ± 2.2	3.6 ± 0.7	4.2 ± 1.0	33.4 ± 7.6	43.0 ± 3.1
		*SL6#		*SL6	*SL6	*SL6 #					*SL6				*SL6	
SL6	8.4 ± 6.8	180.0 ± 2.0	7.0 ± 7.4	3.0 ± 2.8	15.0 ± 1.7	1.5 ± 0.3	26.2 ± 2.9	19.1 ± 3.7	5.7 ± 1.3	7.7 ± 0.5	15.4 ± 8.7	37.8 ± 1.6	6.8 ± 3.6	5.5 ± 0.4	8.6 ± 12.0	32.3 ± 1.8
	*SL0, SL2	*SL4#		#	*SL0, SL4	*SL4 #		#			*SL4	#			*SL4	#

*Note*: The values were expressed as means ± SD.

* The differences between workload groups (SST, LST accordingly) are significant at <0.05 for the Kruskal–Wallis ANOVA test.

^#^ The differences between SST and LST groups are significant at <0.05 for the Mann–Whitney (*U*) test.

The concentrations of glucose and lactate in the blood of rats after exercise to exhaustion generally tended to be higher as the weight of the attached load increased. However, the picture became clearer when the rats were separated within each group according to the duration of swimming. Lactate and glucose levels in LST rats were significantly lower than those in SST rats.

The average values of the other parameters did not show regularities or trends that corresponded with differences in load weights or the duration of swimming in groups without separation. However, the differences became apparent when the groups were divided into SST and LST groups. The concentrations of cortisol were higher in SST rats than in LST rats, and the concentrations of urea were lower. Levels of nitric oxide metabolites were also notable. The NO_x_ index was significantly higher in LST rats than in SST rats. The significant differences in NO_x_ values primarily depended on the levels of NO_3_, and the levels of NO_2_ showed no significant differences between groups.

### 3.1 Anaerobic threshold

**FIGURE 2:**
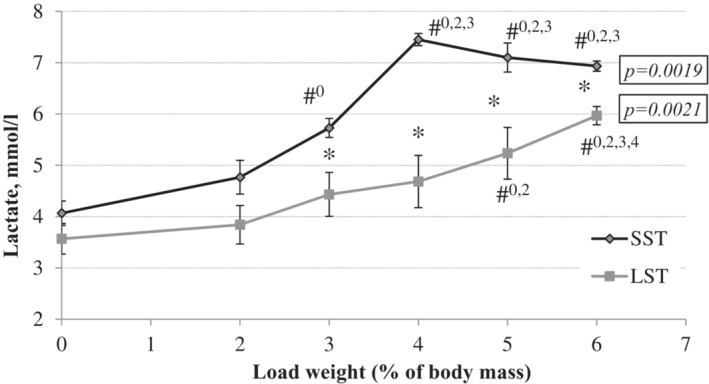
Blood lactate levels in tail vein blood of rats from SST and LST groups. SST—swimming for short time, LST—swimming for long time. The values are expressed as means ± SD. The statistical significance of differences between SST and LST groups was estimated using Mann–Whitney (*U*) test; *p*‐values were considered significant at *p < 0.05. The statistical significance of differences between workload groups within corresponding group (SST or LST accordingly) was estimated using the Friedman ANOVA and Kendall coefficient of concordance, and is shown in italics. When necessary, the Newman–Keuls post hoc comparison test was used. Statistical significance is indicated in comparison with the specified load weight at ^#^ *p* < 0.05.

Based on the time of swimming to exhaustion, all rats were divided into rats that swam for more than 240 min (LST, *n* = 4) and rats that swam for less than 90 min (SST, *n* = 4). The dynamics of lactate and the level of the AT in these groups are shown in Figure 2.

The dynamics of lactate in venous blood differed in these two groups of rats. In the SST group, we observed a sharper increase in this indicator, and starting at the load of 3% BW, we registered a statistically significant difference from the first data point. However, the most significant increase in lactate levels was detected at a load of 4% BW. Further testing of rats at loads of 5%–6% BW did not show significant changes in lactate levels compared with a load of 4% BW. Therefore, the AT in SST rats was assessed at the level of 3% BW. The lactate curve in LST rats had a flatter slope than the SST curve and was characterized by a more gradual increase in the concentration of lactate in the blood with the increasing weight of the attached loads. The most significant increase in this indicator occurred when the rats performed the test at a load of 6% BW. Therefore, the AT for the LST group was set at a load of 5% BW.

The concentrations of lactate, glucose, and cortisol in arteriovenous blood collected after decapitation of rats were higher in SST rats than in LST rats (Table 3).

**TABLE 3:** Biochemical parameters of arteriovenous blood of sacrificed rats after performing the last load (6% BW).

Groups	Lactate, mmol/L	Glucose, mmol/L	Cortisol, ng/mL	Urea, mmol/L	NO_x_, μmol/L	NO_2_, μmol/L	NO_3_, μmol/L
SST	10.3 ± 4.4	9.5 ± 0.8	34.2 ± 9.3	4.0 ± 1.0	28.1 ± 1.8	9.5 ± 1.6	18.6 ± 3.1
LST	6.3 ± 0.7	8.5 ± 0.7	19.1 ± 3.2	4.5 ± 1.1	24.3 ± 1.8	8.0 ± 1.1	16.3 ± 2.9
*p‐*value^SST‐LST^	**0.049**	0.126	**0.049**	0.512	**0.049**	0.126	0.512

*Note*: Data are presented as the means ± SD. *p‐*value^SST‐LST^—The statistical significance of differences between short swimming time (SST) and long swimming time (LST) groups was estimated using the Mann–Whitney (*U*) test. *p* < 0.05 are shown in bold.

Urea levels in the SST and LST groups showed no significant differences. The NO_x_ levels were significantly higher in SST rats than in LST rats. The levels of NO_2_ and NO_3_ did not reveal significant differences in the arteriovenous blood of either group of rats. However, we observed higher values of both metabolites in SST rats than in LST rats.

## 4 DISCUSSION

Only a few studies have described swimming T_ex_ in rats (Beck & Gobatto, 2013; Travassos et al., 2018; Venditti & Di Meo, 1996). Some groups used arbitrary loads without describing the reasoning for weight choice (Travassos et al., 2018; Venditti & Di Meo, 1996). Other researchers used a method in which the load to exhaustion was based on a preliminary calculation of AT. For example, Beck et al. (2014) used the minimum lactate level on the “U‐shaped” lactate curve obtained after the blood LMT as the AT. The loads that the rats were subjected to in this test were then used as benchmarks for the swimming to exhaustion experiment. However, the method of calculating AT significantly affects the interpretation of the results and makes it impossible to compare the results with those of other studies. Therefore, we evaluated T_ex_ in rats swimming to exhaustion using various loads in the first stage of our study (Table 1). Predictably, the increase in the load weight affected the duration of the swim and reduced it from several hours to several minutes. However, the swimming time also strongly depended on the endurance of the rats. For most load weights, T_ex_ was divisible into two groups (Table 2): SST and LST. Loads heavier than 8% BW were too heavy for untrained rats (Gobatto et al., 2001), and no differences were observed in this case. Despite the lack of similar studies in the literature, there are fragmentary results on rats swimming to exhaustion. For example, Venditti and Di Meo (1996) showed a T_ex_ close to our results (294 ± 32 min) in untrained rats (swimming to exhaustion) with a load of 2% of BW. Beck and Gobatto (2013) reported that rats swam 108 ± 46 min with a load weight of 5% BW, which is less than the load of 4%–6% BW in our study. A greater variation in T_ex_ was described in Travassos et al. (2018).

Rats in the Travassos et al. (2018) study swam at a load of 6% BW from 3 to 22 min and were divided into low performance (T_ex_ = 3–12 min) and high performance (T_ex_ = 12–22 min) groups depending on the time of exhaustion. Notably, the authors excluded all rats that swam longer than 22 min. The variability in the duration of swimming with the load of 6% BW was larger in our study, and therefore, the division into endurance groups was different. All rats that swam less than 21 min with a load of 6% BW were included in the SST group, and rats that swam approximately 180 min were included in the LST group. Therefore, the study of untrained rats using various load weight protocols allows comparison of metabolic changes in LST and SST animals and identifies the best load weight for the test.

We showed that the blood biochemistry changes in different load weights and the time of swimming to exhaustion also significantly differed between the LST and SST rats. The SST rats had higher levels of lactate, glucose and cortisol, and the LST rats had higher levels of urea and nitric oxide. Notably, these differences became more obvious with increases in the load weight while swimming.

Despite all of the rats being of the same age, being housed in the same conditions, being fed the same diet, and having no previous training, some rats showed higher inborn physical endurance. These results are fully consistent with the hypothesis that genetics is an important determinant of the response to physical activity (Koch et al., 2005) and may affect the features of anatomy (Britton & Koch, 2001), pulmonary function (Kirkton et al., 2009), insulin response (Schwarzer et al., 2021), and the predominant type of skeletal muscle fibers (Abernethy et al., 1990). It was expected that the metabolism in rats with different physical endurance would be different. A significant increase in blood lactate at the lower loads in SST rats reflects lower aerobic capacities, and hypoxia occurs faster in these rats under high‐intensity physical exercise. Howlett et al. (2009) showed that SST rats had VO_2max_ and oxygen transfer in skeletal muscles that was 50% lower than those in higher endurance rats, despite having higher absolute muscle mass. The maintenance of glucose levels in hypoxia is provided primarily by glycolysis and glycogenolysis (Brooks & Mercier, 1994; Emhoff et al., 2013). With sufficient oxygen supply during prolonged physical exercise, there is a higher fat utilization. The increased contribution of lipids to energy metabolism makes it possible to significantly increase endurance during physical exercise (Brooks & Mercier, 1994; Nosaka et al., 2009). There are more data on a more complex system of regulation of lipid metabolism depending on the intensity of exercise (Lyudinina et al., 2018; Romijn et al., 1993).

Physical exercises also stimulate increases in cortisol levels. This hormone plays a significant role in accelerating lipolysis, ketogenesis, and proteolysis (Del Corral et al., 1998). The level of cortisol increases in proportion to the intensity of exercise, but the final level depends on the total duration of exercise. Moderate‐ and high‐intensity exercises increase the levels of circulating cortisol. In contrast, low‐intensity exercise does not lead to an increase in cortisol levels (Del Corral et al., 1998; Hill et al., 2008).

The levels of cortisol did not show significant differences with respect to T_ex_ in our study. However, cortisol levels were higher in SST rats than in LST rats, especially at loads of 2%–4% BW. Perhaps, this result occurred because of the different behaviors of rats when performing the test and the levels of individual stress. Glucocorticoids in rodents are often used as biomarkers of stress, with cortisol reacting faster during severe acute stress, unlike corticosterone, which is associated more with adaptation during chronic stress (Gong et al., 2015).

NO is another metabolite that allows adaptation to significant physical exercise (Oral, 2021). It is a signaling molecule with a wide variety of effects in mammals, the most well‐known of which is the regulation of local vasomotor tone and resistance to microvascular flow (Baskurt et al., 2011). Skeletal muscles of rodents contain unusually high concentrations of nitrates compared to blood and other tissues, which indicates the high importance of nitric oxide for their body (Piknova et al., 2015). Nitric oxide has an extremely short half‐life of only a few milliseconds in biological tissues, and it is important that it is constantly produced at its sites of effect (Jones et al., 2021). Experimental data indicate that physical exercises lead to an increase in the enzymatic synthesis of nitric oxide and activation of the associated vascular control mechanisms (Baskurt et al., 2011). We previously found a positive correlation between nitrogen oxide and lactate at the AT and a negative correlation at maximum load in elite cross‐country skiers possessing high endurance (Parshukova et al., 2020). The higher level of NO_x_ we obtained in LST rats, but not SST rats, is consistent with these findings. It characterizes a more adequate response of the vascular bed in response to physical exercise and allows better control of vascular tone for a longer time. The increase in NO_x_ levels in LST rats was observed primarily due to the NO_3_ fraction. Under conditions of normal and increased oxygen consumption by tissues, NO is formed enzymatically via the oxidation of L‐arginine, and the final metabolite of this process is primarily NO_3_ (Cubrilo et al., 2011).

Our study showed that the use of a load weight of 4% BW was the most informative for studying the level of physical endurance in untrained rats. At this load weight and a background of a wide T_ex_ range, the rats showed significant changes in most of the biochemical indices assessed in our study, which included the most informative indices in relation to the problem under discussion.

### 4.1 Anaerobic threshold

There are a large number of methods for studying the lactate threshold in rats (Faude et al., 2009). The choice of method depends on the research goals. However, it is also important to consider the natural abilities of rats and our capacity to project the results on humans in the future. The optimal method for determining the AT in rats is swimming with increasing load. Several studies of the lactate threshold in swimming rats found that loads of 4%–6% BW were more often used (Contarteze et al., 2008; Gobatto et al., 2001; Voltarelli et al., 2002). For example, Gobatto et al. (2001) showed that the AT corresponded to 6% BW at a blood lactate concentration of 5.5 mmol/L. Another study established the AT at a weight of 4.0% BW and a lactate level of 5.2 mmol/L (Abreu et al., 2016). Similar lactate values were shown at an AT with a load of 4.5% BW (Zhouab et al., 2018). However, these studies do not mention individual endurance variation in rats. The results of our study showed that this characteristic of laboratory animals may significantly shift the AT to the left or the right on the lactate curve. The ATs in SST and LST rats were 3% BW and 5% BW, respectively. The lactate curve of SST was less flat than that of LST. For increasing endurance, it is generally recognized that a shift of the lactate curve to the right is interpreted as an increase in physical performance, and a shift to the left is considered a deterioration in endurance (Abreu et al., 2016; Faude et al., 2009). The lactate concentration at the AT was also different and higher in SST rats than in LST rats (5.8 mmol/L vs. 5.2 mmol/L). A lower lactate level at the end of physical exercise in LST rats may be associated with a lower rate of lactate accumulation and/or a lower metabolic clearance of lactate (Donovan & Brooks, 1983; Yang et al., 2020). Higher endurance augments capacities for lactate production, disposal, and clearance (Messonnier et al., 2013). Our data are generally consistent with the results of other studies, although no data on the AT when swimming in rats with a load below 4% were found. However, rats with higher endurance were likely included for various reasons in the described studies (Abreu et al., 2016; Contarteze et al., 2008; Gobatto et al., 2001; Voltarelli et al., 2002; Zhouab et al., 2018).

Biochemical data from the arteriovenous rat blood assays (Table 3) also demonstrated significant differences between the SST and LST rats. Because the collection of arteriovenous blood occurred within 3–5 min after the last collection of blood from the caudal vein, the data on lactate from arteriovenous blood showed higher values relative to lactate from the caudal vein. The most significant increase was observed in SST rats, which reflected their lower recovery abilities compared to LST animals. Glucose, cortisol, and NO_x_ levels were also significantly higher in the SST rats. This pattern of blood biochemistry generally characterizes more significant rearrangements and higher stress levels in SST rats than in LST rats at a similar level of physical exercise. Notably, the increase in NO_x_ levels in this test occurred due to an increase in nitrites (NO_2_), unlike in swimming to exhaustion. Under hypoxic conditions, NO_2_ is an alternative source of nitric oxide synthesis (Gladwin et al., 2000; Schulman & Hare, 2012) and participates in adaptation to hypoxia caused, for example, by physical exertion (Gladwin et al., 2000). The current understanding of nitrite‐dependent mechanisms of adaptation to hypoxia is based on data on the reduction of NO_2_ by oxygen‐dependent and hypoxic nitrite reductase (Gladwin & Kim‐Shapiro, 2008).

NO is a mediator of skeletal muscle function and affects cellular respiration and contractility. In working skeletal muscle, inhibition of NOS improves the economy of muscle contraction, decreases the outflow of lactate from the muscles, and reduces the oxygen cost (Krause & Van Etten, 2005). Thiol groups, reactive metal ions in the proteins' active centers, can interact with NO, which leads to various responses and further biological events in skeletal muscles. NO‐mediated reactions inhibit heme‐containing proteins, such as cytochrome C oxidase, thus interfering with the function of cytochrome C oxidase in cell respiration (Borutaite & Brown, 1996). Inhibition of this enzyme and of the sarcoplasmic reticulum Ca^2+^‐ATPase in fast‐twitch and slow‐twitch skeletal muscle fibers by NOS‐generated NO may also lead to inhibition of mitochondrial respiration in skeletal muscle (Klebl et al., 1998). Moreover, aconitase and respiratory chain complex I can also be targeted by NO (Clementi et al., 1998). NO is crucial for the activation and inhibition of ryanodine receptors (RyRs) (Stamler & Meissner, 2001), which play a decisive role in the release of Ca^2+^ into the cytosol and therefore in muscle excitation and contraction (Mazzone & Carmeliet, 2008). In our experimental work, we have shown that elite athletes (cross‐country skiers) have an NO‐dependent mechanism for regulating lactate levels during aerobic exercise, especially when working at the AT. In particular, at the AT, we have revealed a positive relationship between NO_x_ (nitric oxide metabolites) and blood lactate, with that relationship being reversed at maximum load. This observation suggests the existence of an adaptive mechanism for regulating lactate levels at the AT in highly qualified cross‐country skiers (Parshukova et al., 2022).

Therefore, our data provide a new understanding of the role of NO‐dependent mechanisms in the phenomenon of AT.

## 5 CONCLUSION

We found that the level of individual endurance significantly affected the AT in untrained rats. The AT in SST rats and 5% BW in LST rats. These groups also had different blood biochemistry profiles at the AT and after swimming to exhaustion. There was a shift in the AT to the right side on the lactate curve in the zone of the AT in LST rats compared to SST rats, and the levels of lactate, glucose, cortisol, and NO_x_ were lower. At the end of the exercise to exhaustion, SST rats had higher blood levels of lactate, glucose, and cortisol, and LST rats had higher levels of urea and NO_x_.

## AUTHOR CONTRIBUTIONS

Evgeny Bojko, Nadezhda Vakhnina, Natalya Potolitsyna conceives and designed the experiments; Natalya Potolitsyna, Nadezhda Vakhnina, Nadezhda Alisyltanova, Lubov Kalikova, Anastasia Tretyakova, Alexey Chernykh, Vera Shadrina, Arina Duryagina performed experiments; Natalya Potolitsyna, Olga Parshukova, Lubov Kalikova analysed data; Natalya Potolitsyna, Olga Parshukova, Lubov Kalikova, Nadezhda Vakhnina interpreted results of experiments; Natalya Potolitsyna prepared figures; Natalya Potolitsyna, Alexey Chernykh drafted manuscript; Natalya Potolitsyba, Evgeny Bojko, Olga Parshukova, Nadezhda Vakhnina, Alexey Chernykh edited and revised manuscript; Natalya Potolitsyna, Evgeny Bojko, Olga Parshukova, Nadezhda Vakhnina, Alexey Chernykh approved final version of manuscript.

## FUNDING INFORMATION

This research did not receive any specific grant from funding agencies in the public, commercial, or not‐for‐profit sectors.

## CONFLICT OF INTEREST STATEMENT

No conflicts of interest, financial or otherwise, are declared by the authors.

## ETHICS STATEMENT

The Ethics Committee of the Institute of Physiology of the Russian Academy of Sciences, Syktyvkar approved the experimental design and protocol of the study. The study was performed in accordance with the ethical standards as laid down in the 1964 Declaration of Helsinki and its later amendments.
